# Supplementary material for: Impacts of Human Activity and Climate Change on the Suitable Habitats for Xanthium spinosum in China
Source: Plants (Basel). 2025 Jan 21;14(3):306. doi: 10.3390/plants14030306 (PMC11819725; doi:10.3390/plants14030306)
Supplement: Supplementary file 1 [file plants-14-00306-s001.zip › Table S2.pdf]

Supplementary material

Table S2. 34 Environmental variables (bold indicating the variables used for modeling:16)

Type of variables	Environmental variables (Code)
Climatic factors	(°C) Annual mean temperature (bio1)
	(°C) Mean diurnal range (bio2)
	**Isothermality (bio3)**
	Temperature seasonality (bio4)
	**(°C) Maximum temperature of the warmest month (bio5)**
	(°C) Minimum temperature of the coldest month (bio6)
	**(°C) Temperature annual range (bio7)**
	**(°C) Mean temperature of the wettest quarter (bio8)**
	**(°C) Mean temperature of the driest quarter (bio9)**
	(°C) Mean temperature of the warmest quarter (bio10)
	(°C) Mean temperature of the coldest quarter (bio11)
	**(mm) Annual precipitation (bio12)**
	(mm) Precipitation of the wettest month (bio13)
	**(mm) Precipitation of the driest month (bio14)**
	**Precipitation seasonality (bio15)**
	(mm) Precipitation of the wettest quarter (bio16)
	(mm) Precipitation of the driest quarter (bio17)
	**(mm) Precipitation of the warmest quarter (bio18)**
	**(mm) Precipitation of the coldest quarter (bio19)**
Terrain factors	**(m) Elevation (elev)**
	Slope (SLO)
	**Aspect (Asp)**
Human impact index factors	**Human impact index (HII)**
Soil factors	Organic carbon (ORG_CARBON)

Type of variables	Environmental variables (Code)
	Soil bulk density (REF_BULK)
	**Clay content (CLAY)**
	Sand content (SAND)
	**Silt content (SILT)**
	Coarseness (COARSE)
	Cation exchange capacity (CEC_SOIL)
	**PH (PH_WATER)**
	Total nitrogen (TOTAL_N)
	C/N ratio (CN_RATIO)
	Electrical conductivity (ELEC_COND)

Table S3. Selection of the model threshold

Model	Threshold
Ensemble model	Cutoff (733≈700)
